# Screening Readthrough Compounds to Suppress Nonsense Mutations: Possible Application to β-Thalassemia

**DOI:** 10.3390/jcm9020289

**Published:** 2020-01-21

**Authors:** Monica Borgatti, Emiliano Altamura, Francesca Salvatori, Elisabetta D’Aversa, Nicola Altamura

**Affiliations:** 1 Biotechnology Center, University of Ferrara, 44121 Ferrara, Italy; monica.borgatti@unife.it (M.B.); francesca.salvatori@unife.it (F.S.); elisabetta.daversa@unife.it (E.D.); 2 Chemistry Department, University of Bari, 70126 Bari, Italy; emiliano.altamura@uniba.it; 3 Institute of Biomembranes, Bioenergetics and Molecular Biotechnologies, National Researches Council, 70126 Bari, Italy

**Keywords:** nonsense suppression, premature termination codon, nonsense mediated mRNA decay, β^0^-thalassemia, readthrough molecules

## Abstract

Several types of thalassemia (including β^0^39-thalassemia) are caused by nonsense mutations in genes controlling globin production, leading to premature translation termination and mRNA destabilization mediated by the nonsense mediated mRNA decay. Drugs (for instance, aminoglycosides) can be designed to suppress premature translation termination by inducing readthrough (or nonsense suppression) at the premature termination codon. These findings have introduced new hopes for the development of a pharmacologic approach to cure this genetic disease. In the present review, we first summarize the principle and current status of the chemical relief for the expression of functional proteins from genes otherwise unfruitful for the presence of nonsense mutations. Second, we compare data available on readthrough molecules for β^0^-thalassemia. The examples reported in the review strongly suggest that ribosomal readthrough should be considered as a therapeutic approach for the treatment of β^0^-thalassemia caused by nonsense mutations. Concluding, the discovery of molecules, exhibiting the property of inducing β-globin, such as readthrough compounds, is of great interest and represents a hope for several patients, whose survival will depend on the possible use of drugs rendering blood transfusion and chelation therapy unnecessary.

## 1. Introduction

Gene expression requires quality control mechanisms to ensure the synthesis of fully functional gene products. Many errors produced during the various steps of gene expression may cause genetic diseases. A significant subset of such errors consists of nonsense mutations, frameshift mutations, and mutations that result in alternative splicing events that interrupt the open reading frames by introducing premature termination codons (PTCs). Recent advances of genomic technologies, allowing complete inspection of the genome sequence and genome editing, constitute an unprecedented opportunity to diagnose genetic disorders and pave the way to gene therapy. It has been estimated that approximately 12% of inherited genetic diseases and several forms of cancer are caused by nonsense mutations, resulting in a single nucleotide change that converts a sense codon within protein coding genes into a premature termination codon [1]. PTCs (or nonsense mutations) are generally associated with a dramatic reduction in gene expression. mRNAs containing in-frame PTCs (PTC-mRNAs) are substrates of the nonsense mRNA mediated decay pathway (NMD), a mRNA surveillance process that recognizes and rapidly degrades them. PTCs, therefore, preclude the synthesis of a functional full length protein because the expression of PTC genes is compromised by a premature arrest of translation and low level of PTC mRNAs, leading to severe inherited diseases. These include cystic fibrosis (CF) [2], Duchenne muscular dystrophy (DMD) [3], spinal muscular atrophy [4], hemophilia, [5], Hurler syndrome [6], Usher syndrome [7], ataxia telangiectasia [8], lysosomal storage diseases [9], and several genetic diseases and cancer forms recently described. So far, no gene therapy is available for the treatment of these disorders, and the applications of genome editing tools are currently at the level of the disease model organism and are approaching the clinic for some treatments. In recent years, an alternate therapeutic approach, known as “nonsense suppression therapy”, was developed to restore un-functional or aberrant PTC containing genes by using chemical compounds to reactivate gene expression [2,10,11]. Gene dysfunction rescue is based on the ability of certain molecules to overcome the premature arrest of translation by promoting nonsense suppression (readthrough) at PTCs, restoring the full length protein synthesis. The extent of PTC suppression depends not only on the efficacy of the readthrough mediating molecule, but also on the PTC mRNA abundance, which depends on the efficiency of NMD. Inhibition of NMD is therefore considered an important target to facilitate gene expression, and current strategies envisage extensive searching of chemical compounds targeting either premature translation termination, or NMD, or both. In recent years, excellent reviews have been published that cover the huge amount of information derived from the rapid progress in knowledge about (a) the fundamental steps of gene expression like translation [10,11] and NMD [10,11,12,13,14,15,16,17,18], (b) the nonsense suppression therapeutic approach [2,10,11,12,19,20], (c) genetic diseases associated with nonsense mutations linking together the two processes [20,21,22,23], and (d) small molecules able to interfere with one, the other, or both processes [24,25,26,27], in an effort to correct in the cytoplasm errors produced in the nucleus. So far, one of the most widespread genetic diseases, β^0^-thalassemia, caused by nonsense mutations, has not been involved in the therapeutic approach based on nonsense suppression mediated by small molecules, despite it having been a long time since it was shown to be caused by nonsense mutations [28,29,30]. In the present review, we summarize the principle and current status on the chemical relief for the expression of functional proteins from genes otherwise unfruitful for the presence of nonsense mutations, as well as emerging strategies to identify novel safe compounds suitable for combinatorial approaches aimed at suppressing concomitantly premature translation termination and inhibiting NMD. As a novelty for a review in the field, we report examples unraveling the potential of ribosomal readthrough as a therapeutic approach for the treatment of β^0^-thalassemia. Screening small molecules able to induce nonsense suppression in β-globin, as well as NMD inhibitors is of great interest and represents a hope for several patients, whose survival will depend on the possible use of drugs rendering blood transfusion and chelation therapy unnecessary.

## 2. Overview of the Nonsense Suppression Therapeutic Approach

The synthesis of a protein results from mRNA translation. This process initiates when the start codon AUG enters the A-site of the ribosome, with a complimentary base pairing of aminoacyl-tRNA anticodon, loaded with methionine. For each of 61 codons specifying an amino acid, a corresponding aminoacyl-tRNA anticodon is available in the cell (see for details [10,11] and the references therein). During elongation, mRNA shifts such as the AUG codon moving from the A-site (aminoacyl) to the P-site (peptidyl) of the ribosome and an additional aminoacyl-tRNA, loaded with another amino acid, can base pair with the next codon. The latter amino acid binds the methionine, forming a peptide bond. This process is reproduced for each codon along the mRNA sequence until one of the three stop codons UGA, UAG, or UAA enters the A-site. Decoding of the stop codons does not occur via nucleoside base pairing since no complimentary tRNA anticodon is available, and the recognition of each termination codon for translation termination is mediated by eukaryotic translation termination factors, eRF1 and eRF3 (release factors). Occasionally, sampling at the stop codon results in a near-cognate aminoacyl-tRNA replacing eRF1, leading to stop codon suppression (also known as readthrough). This phenomenon does occur in eukaryotes at natural termination codons with a frequency of less than 0.1% and with the efficiency rank UGA > UAG > UAA. Thus, readthrough occurs as a natural process at the normal stop codon when during the proofreading stage, an aminoacyl-tRNA base pairing successfully competes with the release factor eRF1, resulting in insertion of an amino acid in the growing peptide. The expression of several genes has been found to be regulated by readthrough in human cells [20,22]. A premature termination codon (PTC), generated by a single point mutation in a sense codon, is recognized by entering the A-site in the ribosome as in translation termination of the natural stop codon. However, the translation machinery is believed to be endowed with molecular mechanisms able to recognize a PTC as an atypical stop codon. Events involving factors and interactions mainly occurring downstream of the pre-terminating ribosomes stalling at the PTC culminate in the activation of NMD, the process by which mRNAs harboring the premature termination signal are recognized and rapidly degraded [20,31,32,33]. NMD is triggered only when elongating ribosomes sense the presence of PTC internally to the open reading frame located, likely, in a translation termination context thought to be different from the natural stop codon. Several pieces of evidence indicate that translation and NMD are intimately linked processes. For instance, translation is required for PTC recognition since translation inhibitors like cycloheximide, puromycin, or emetine also inhibit NMD. However, the efficiency in translation termination of PTC is lower than natural translation termination and is generally associated with higher susceptibility to suppressing the PTC, i.e., a ten fold increased capability of an aminoacyl-tRNA to be inserted at PTC, compared to the natural stop codon. As a result, an amino acid is added to the elongating peptidyl-tRNA (basal readthrough ≥ 1%).

Several models have been proposed to explain the mechanistic differences between natural translation termination and premature translation termination that triggers NMD in the latter, but not in the former. A description of these models is beyond the scope of this review, and readers should refer to the excellent review by He and Jacobson [17].

Central to all proposed NMD models is the core surveillance complex of NMD composed by three “up frameshift proteins” (Upf)s, Upf1, Upf2, and Upf3. interacting in the sequence Upf1-Upf2-Upf3 in the activation of NMD [25,26,27,28,29,30,31,32,33]. Each of these proteins is required for NMD function. Among them, Upf1, a superfamily I helicase with ATPase and mRNA binding properties [34,35,36], likely plays a pivotal role in communicating the translation termination status at PTC to the mRNP configuration downstream PTC, preluding NMD. First, Upf1 interacts with the release factor eRF1 on the ribosome and then with Upf2-Upf3 associated with mRNA protein complexes downstream of the stalling pre-terminating ribosome. In their unified model, He and Jacobson [17] proposed that premature translation termination is a complex process requiring several functions delivered by the three Upf proteins, covering increased activity of the release factor and the dissociation and recycling of premature terminating ribosomes. Multifunctional Upf1 is subject to a phosphorylation/dephosphorylation cycle, is the principal regulator of NMD, and provides its ATPase activity to promote the key conformational and compositional changes of the terminating mRNP status to initiate and carry it to the completion of NMD. Alternative models for the function of Upf1 have been recently proposed [34,35,36], and new concepts about a more general context into which NMD would operate in gene expression are emerging [14,16,31,37,38,39].

Unravelling the mechanisms governing NMD is a crucial step in identifying targets and factors aimed at attenuating NMD, thus increasing the abundance of PTC mRNAs available for the nonsense suppression therapy.

**Figure 1:**
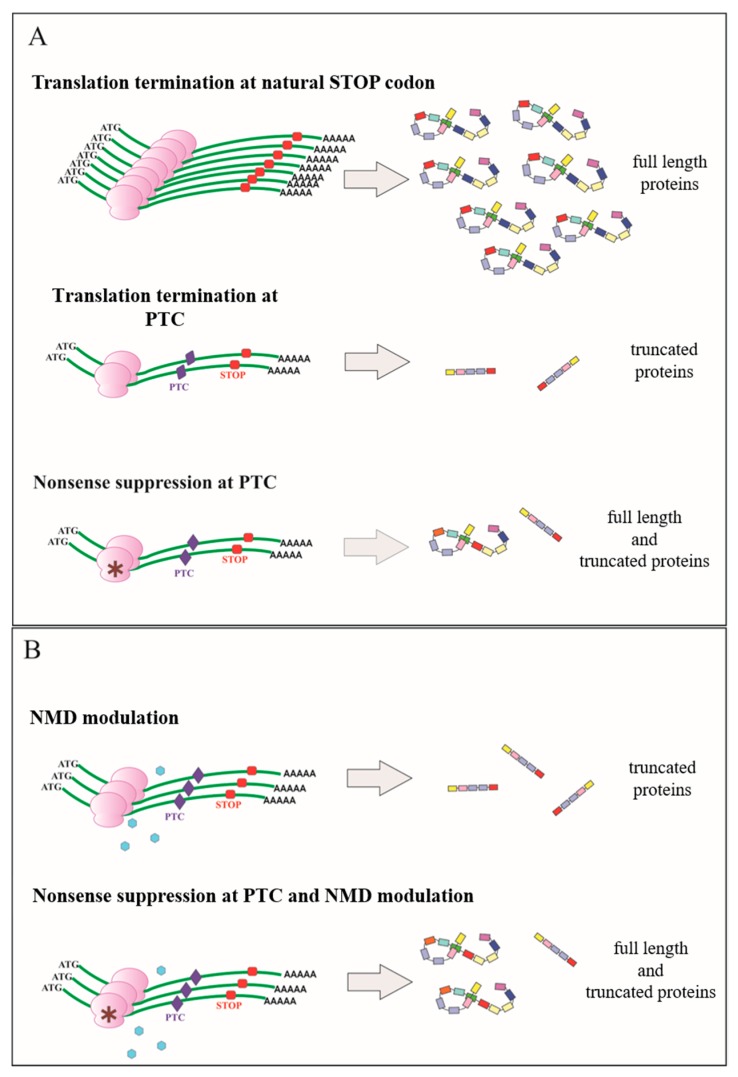
Functional consequences of the premature termination codon (PTC) on gene expression and the basic principle of the nonsense suppression therapeutic approach. (**A**) Natural abundance of physiological mRNA and relevant full length protein production are drastically reduced in the presence of a PTC. The premature arrest of translation results in the synthesis of truncated protein. Nonsense suppression at PTC restores to some extent full length protein synthesis. (**B**) Negative modulation of the nonsense mRNA mediated decay pathway (NMD) attenuates mRNA destabilization and increases mRNA abundance. The concomitant presence of a compound promoting nonsense suppression allows more full length protein production.

Most of the genetic disorders caused by nonsense mutations are characterized by a severe phenotype resulting from the combined effect of the premature arrest of translation and the accelerated degradation of the relevant mRNA. In principle, conditions leading to a decrease in the efficiency of translation termination at PTC could favor, to some extent, the synthesis of a full length protein and the stabilization of mRNA encoding it. Considering that even a small amount of the gene product (1–5% of the normal level) could be sufficient to alleviate the phenotype of a genetic disease [40], a favorable approach is the modulation of the efficiency of the translation termination [2]. Although translation termination is an accurate process, an error can arise when a near-cognate aminoacyl-tRNA with an anticodon is complimentary to two of the three nucleotides in the stop codon UGA, UAG, or UAA. Mispairing of the near-cognate aminoacyl tRNA at one of the stop codons can result in insertion of an amino acid overcoming the translation termination [11]. As an example, a near-cognate codon AAA with a single mispairing respect to the stop codon UAA can incorporate the amino acid Lys. On this basis, a set of amino acids can be preferentially available for the suppression of each stop codon. Very recently, amino acids inserted at UGA, UAG, and UAA have been identified during readthrough mediated by non-aminoglycoside PTC124 in both yeast and human cells and by aminoglycoside G418 (geneticin) in human cells harboring reporters expressing mutated forms of CFTR outlining CF genetic disease (see below) [41,42,43]. Because nonsense suppression occurs at a PTC with a frequency much higher than a normal stop codon, efforts have been made to discover small molecules with the properties of decreasing the fidelity of translation termination at PTCs, but not at the natural stop codon, and possibly of attenuating NMD (Figure 1) [2,11].

## 3. Compounds with Nonsense Suppression Properties

### 3.1. Aminoglycosides

**Figure 2:**
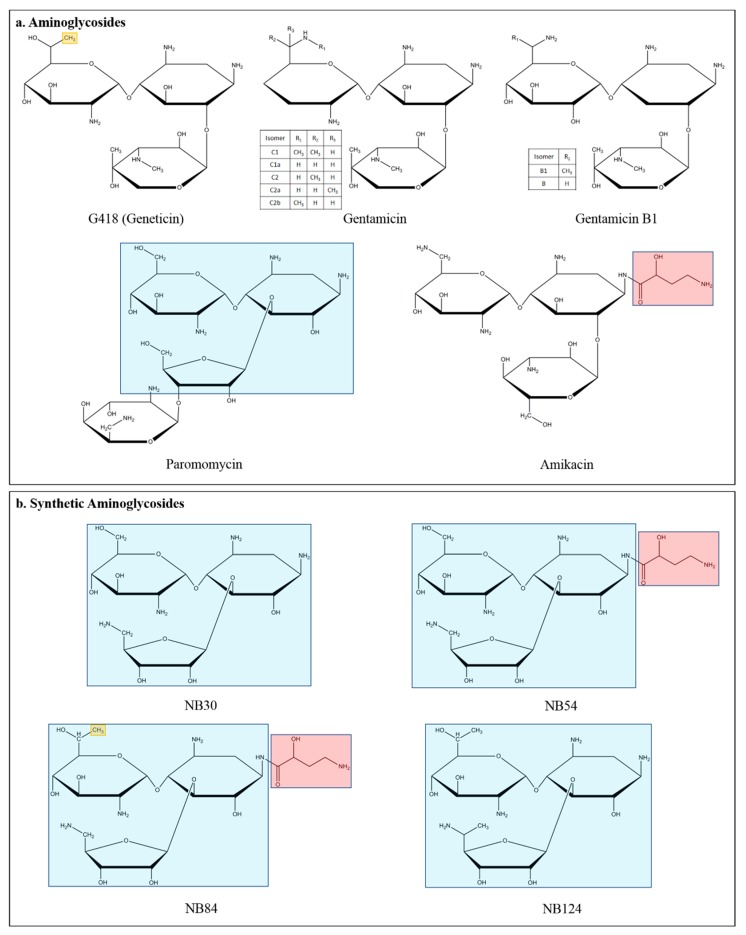
Chemical structures of aminoglycosides mediating PTC suppression. (**a**) Structures of antibiotic aminoglycosides: G418 (geneticin), gentamicin with its isomers, the two isomers of gentamicin B1, paromomycin, and amikacin. (**b**) Synthetic novel designer aminoglycosides: structural features of natural aminoglycosides paromomycin (the three ring pseudo-trisaccharide backbone, in blue), amikacin (functional group called AHB on C10, in light red), and G418 (methyl group on C6′, in yellow) were combined to produce designer aminoglycosides NB30, NB54, NB84, and NB124 [11,24].

The properties of aminoglycoside antibiotics to mediate phenotypic suppression of nonsense mutations were first evidenced in the yeast *Saccharomyces cerevisiae* [44,45]. These compounds, consisting of a 2-deoxystreptamine ring linked to amino sugars (Figure 2), bind the ribosome at the decoding center where the proofreading takes place to select the appropriate cognate aminoacyl-tRNAs. A crucial difference in two nucleotides in the eukaryotic RNA ribosomal sequence, compared to the prokaryotic sequence, strongly reduces the aminoglycoside affinity for the eukaryotic decoding center, thus allowing their use as antibiotics. The first example of nonsense suppression therapy was provided by using the aminoglycoside G418 (geneticin) (Figure 2), in cultured cells harboring nonsense mutations in the cystic fibrosis transmembrane conductance regulator (CFTR), whose dysfunction in humans causes the relevant genetic inherited disease (CF) [46]. Since then, many studies demonstrated the ability of aminoglycosides G418, paromomycin. and gentamicin to promote nonsense suppression in many disease model systems [11,47]. In clinical applications, gentamicin was used in CF and DMD (Duchenne muscular dystrophy) patients with restoration of a significant amount of functional CFTR protein or dystrophin. However, only half of CF patients and fractions of DMD patients exhibited the functional rescue of the CFTR and dystrophin respectively. In addition, long term treatment with aminoglycosides, administered in nasal droplets or intravenously, lead to serious side effects involving hearing loss and nephrotoxicity, although some of these effects could be attenuated by antioxidants (D-methionine, melatonin) and liposomal vehicle administration of aminoglycoside. The cellular target of aminoglycoside antibiotics in eukaryotes is likely the translation machinery, potentially involving the mitochondrial translation system regarding toxicity due to the similarity with the bacterial translation apparatus [48,49,50,51]. Based on the hypothesis that nonsense suppression properties and toxicity are separate functions in the aminoglycoside structure, a rational design strategy was developed by which the moieties responsible for the cytoplasmic binding of the drug were enhanced and the one responsible for mitochondrial binding reduced. The resulting paromomycin derivative aminoglycoside NB30 (Figure 2) was indeed found to be over ten fold less toxic than the original compound while maintaining nonsense suppression ability, although with reduced efficiency compared to gentamicin. Subsequent applications of such a drug re-designing approach led to synthetic compounds based on the modification of neomycin, kanamycin analogs, and a derivative of paromomycin and geneticin (G418) [52]. Among these, the aminoglycoside derivative NB54 (Figure 2) was demonstrated to be much less toxic and more efficient than gentamicin in suppressing nonsense mutations in cell culture and mouse models of diseases like CF, DMD, and the lysosomal storage disease (LSD) mucopolysaccharidosis I-Hurler (MPS I-H) [53] and in cultivated cells harboring nonsense mutations associated with Rett syndrome and Usher syndrome [54,55,56]. A significant improvement in the suppression of nonsense mutations related to MPS I-H, in mouse models, was achieved with NB84 (Figure 2), a further version of modified aminoglycoside proven to be superior to gentamicin [57,58]. A new generation of synthetic aminoglycoside, NB124 (Figure 2), was demonstrated to be more efficient than gentamicin in suppressing the nonsense mutations G542X, R1162X, and W1282X, among the prevalent nonsense mutations in the CFTR gene causing CF. NB124 was found able to restore the full length synthesis of CFTR and chloride transport in an animal genetic model and rescue about 7% of CFTR function in primary human bronchial epithelial (HBE) CF cells [59]. Most recently, NB124 was identified as a potent nonsense suppressor of several nonsense mutations located in the p53 and APC (adenomatous polyposis coli) tumor suppressor genes, which account for 10% and 30% of mutations in human cancers, respectively [60]. NB124 restores the full length expression of p53, which is functional in inducing the transcription of its target genes. Remarkably, the nonsense suppression efficiency of NB124 supersedes gentamicin in efficacy by ten fold.

New pseudotrisaccharide derivatives of aminoglycosides, in addition, have been designed that exhibit enhanced readthrough properties, compared with gentamicin, on mutations underlying the genetic diseases CF, Usher syndrome, and Hurler syndrome [61]. Overall, this approach proved to be convincing in the development of novel safe and efficient compounds useful for the nonsense suppression therapy of various diseases. However, recent findings have shown that gentamicin, the major component of the pharmaceutical aminoglycosides mixture, often administered as “gentamicin” to humans, lacks PTC readthrough properties, whereas gentamicin B1, present as a minor component of the pharmaceutical gentamicin aminoglycosides’ mixture, demonstrated, when purified, potent PTC readthrough capabilities at all three UAG, UGA, and UAA PTCs in several cultured cell models expressing various nonsense alleles. Furthermore, gentamicin was found to suppress gentamicin B1 readthrough ability [62].

**Figure 3:**
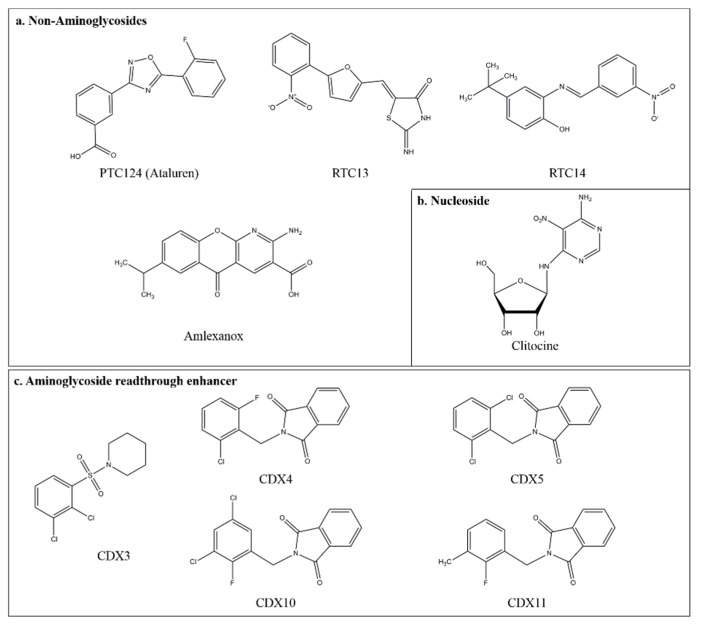
Chemical structure of compounds with nonsense suppression properties and aminoglycoside readthrough enhancer. (**a**) Structures of non-aminoglycosides that induce PTC suppression. Non-aminoglycoside compounds, such as ataluren (PTC124), RTC13, RTC14, and amlexanox, are small organic molecules with no structural similarity to aminoglycosides. (**b**) Structure of clitocine, a naturally occurring adenosine nucleoside analog. (**c**) Structures of the CDX compounds that are aminoglycoside readthrough enhancers, identified in the high throughput screen.

A novel promising approach was recently described that was based in a high throughput screening for molecules able to potentiate the nonsense suppression mediated by aminoglycosides. Five compounds, named CDX3, CDX4, CDX5, CDX10, and CDX11 (Figure 3), were identified that were not able to mediate readthrough in human cells when used alone, but strongly enhanced the readthrough ability of aminoglycosides [63]. The most potent of the novel compounds, CDX5, was found to greatly increase the G418 mediated readthrough (up to 180 fold) with respect to G418 used as a single agent. These findings indicated that a much lesser amount of G418 could be used for an efficient readthrough with minor toxic effects. A cooperative effect in the aminoglycoside mediated readthrough was also observed for poly-L-aspartic acid. This polyanion was previously described as a protective agent against alterations induced by aminoglycosides in cultured human proximal tubule cells, as well as in gentamicin induced early renal alterations. In a more recent study, an increase (up to 40%) of nonsense suppression mediated by gentamicin, in the presence of poly-L-aspartic acid, was detected in a nonsense mouse model of CF, in which CFTR was more functional than with gentamicin alone ([10] and the references therein). Synergistic effects were obtained both in vitro and in vivo studies in nonsense mutations’ readthrough combining ivacaftor, a gating-channel potentiator, with the synthetic aminoglycoside NB124 [59]. Very recently, a CF patient homozygous for the nonsense mutation W1282X, a C-terminal CFTR mutation that results in a truncated protein with partial activity, was subjected to treatment with ivacaftor, resulting in therapeutic benefit. Combining ivacaftor with aminoglycoside G418 in W1282X expressing cells derived from the same patient further increased CFTR activity [64].

### 3.2. Ataluren (PTC124)

In the search for small molecules that could have readthrough properties, be safe, and orally administered, high throughput screens of 800,000 compounds were performed by using luciferase reporters stably expressed in HEK293 cells or rabbit reticulocytes [65]. Subsequent characterization studies led to identifying a small molecule drug ataluren (3-(5-(2-fluorophenyl)-1,2,4-oxadiazol-3-yl)-benzoic acid; also known as PTC124 or Translarna [2]. The structure of ataluren (Figure 3) is different from aminoglycoside antibiotics (Figure 2), has no antibiotic properties, and has no similarity to other clinically available compounds. Importantly, ataluren is efficient at suppressing all three stop codons, in the hierarchy UGA > UAG > UAA, but not the natural stop codon, and displays maximal activity at a concentration much lower (3 μM) than gentamicin when used for comparison. Following an extensive pharmacological characterization, ataluren was tested with many disease models based on the general concept that a single readthrough inducing molecule could alleviate the phenotype of different diseases when each of them is associated with nonsense mutations. Initial preclinical studies on ataluren’s potential in nonsense suppression therapy were related to Duchenne muscular dystrophy DMD, a rare genetic disorder caused by mutations in the gene for dystrophin. Patients unable to express this muscle protein loose ambulation from childhood or early adolescence. Ataluren treatment of *mdx* mouse cultured cells or *mdx* mice harboring a UAA PTC in the dystrophin gene showed the production of full length dystrophin protein and enhanced muscle function respectively. In cystic fibrosis (CF), another severe inherited disease caused by loss of function of the gene encoding the cystic fibrosis trans-membrane conductance regulator CFTR, approximately 10% of cases are caused by nonsense mutations. In the CF mouse model expressing a human CFTR with a nonsense mutation, treatment with ataluren restored the synthesis of a functional CFTR [66]. The ataluren approach in clinical studies (phase 1, phase 2a) demonstrated pharmacological activity in CF and DMD patients. Consistent with results in model systems, ataluren was shown to induce the synthesis of full length CFTR protein CF and increase the expression of dystrophin in a significant fraction of DMD patients. Importantly, the restored CFTR protein was functional in CF patients, and a three months treatment with ataluren recovered even a better pulmonary function [67,68,69,70]. Subsequent international clinical trials for DMD (phase 2b) and CF (phase 3) assessed that ataluren was well tolerated, exhibiting a safety profile similar to placebo. In DMD patients, a positive trend in improvement of muscle function, as well as other measured physical functions was observed after 48 weeks of ataluren treatment. In CF clinical trials, ataluren treated patients were not differentiated from the placebo controls concerning the lung function. CF patients were under simultaneous antibiotic chronic therapy with, among others, aminoglycoside tobramycin, which in further analysis was indicated as an antagonist of ataluren. Patients who were not inhaling tobramycin showed indeed alleviation of pulmonary exacerbations. An extension of the phase 3 clinical trial was launched for CF patients not administered with tobramycin therapy. Having shown that ataluren helps slow the disease progression, this drug has received a conditional approval from the European Medicines Agency for ambulatorial treatment of DMD patients ≥5 years old. Overall, unless with some cell systems [71,72], ataluren has been shown to rescue the dysfunction associated with PTCs in many types of nonsense reporter and mouse disease model systems, in cultured human cells, and human patients. In addition, ataluren was demonstrated to be improved by altering its structure on the basis of a computer driven re-designing approach [73]. However, despite the huge amount of evidence about ataluren’s readthrough potential, very recent outcomes of the extended phase 3 study in CF patients did not achieve its primary and secondary endpoints. Inconsistent results for PT124 in readthrough assays have also been recently reported [74,75]. These controversial issues about the readthrough potential of PTC124 deserve a wide analysis about the genetic and physiological context in which this molecule is operating (see the Conclusion). A crucial point in nonsense suppression therapy is that the amino acid incorporated during readthrough may differ from the original and affect the natural function of the protein. In recent studies, the nature of the translational events mediated by ataluren, and aminoglycosides G418 and gentamicin, has been investigated in the simple eukaryote *Saccharomyces cerevisiae*, as well as in human cells expressing multiple nonsense mutations including a CFTR nonsense allele [42,43]. Readthrough products expressed by specifically constructed reporters were purified and identified by MS in both systems and shown to contain amino acid insertions at PTCs analogous to those observed in the basal readthrough. When the PTCs were UAA or UAG, Gln, Lys, or Tyr was inserted, whereas at UGA PTCs, Trp, Arg, or Cys was inserted, demonstrating that ataluren stimulates selection of near-cognate tRNAs preferentially through mRNA:tRNA mispairing at positions 1 and 3. Importantly, each replacement was functional, suggesting it is unlikely that the readthrough products could acquire immunogenicity or dominant negative functions [39]. In a very recent study, it was shown that an identical set of amino acids, Trp, Arg, and Cys, was incorporated during readthrough mediated by G418 at the UGA stop codon [41], indicating that chemically induced readthrough mimics the basal readthrough in yeast [76]. However, aminoglycoside mediated nonsense suppression and the efficiency of translation termination are known to depend on the local mRNA sequence [77,78]. By using reporters expressing the CFTR mutation W1282X (UGA) embedded in its surrounding context and subjected to nonsense suppression by G418, the set of amino acids Leu (58%), Cys (38%), and Trp (4%) was identified [42]. Surprisingly, these amino acids differ, both in identity and proportions, from those identified for the CFTR G542X mutation, another UGA PTC, including Cys (44%), Trp (36%), and Arg (20%), in the previous study [43]. These results shed light on the mechanism of nonsense suppression, both in the basal and chemical mediated conditions, and add predictive value about the quality of the amino acid inserted during the readthrough as a function of the mRNA local context surrounding PTCs.

### 3.3. Novel Small Compounds with Nonsense Suppression Properties

Novel non-aminoglycosides with readthrough properties have been identified, by using an ELISA based assay, in a screening of a library of 34,000 small compounds for searching for those capable of suppressing nonsense mutations in the ATM gene that causes ataxia telangiectasia (AT) [79]. Two compounds, RT13 and RT14, were most effective at restoring ATM kinase function in AT patient fibroblasts [79]. The same compounds were subsequently found to relieve the expression of the full length dystrophin protein in myotubes derived from a nonsense mouse model of DMD [80]. Additional antibiotic compounds including macrolide [81,82] and negamycin peptide antibiotics were identified to promote readthrough in mice and skeletal muscle *mdx* models [83,84]. In another approach, the ability to induce readthrough was screened among a collection of 1200 small molecules already in use in the clinic for other purposes. This screening led to the identification of amlexanox (Figure 3), found to act as a suppressor of nonsense mutations in human cell disease models [85]. In addition, the anti-inflammatory amlexanox was also shown to suppress multiple disease associated nonsense mutations in mammalian cells [85]. The approach of re-designing molecules recently led to generating nontoxic derivatives of PTC124 in which the fluoroaryl moiety was altered. The novel PTC124 derivatives resulted in being more potent than PTC124 in the suppression of a premature termination UGA codon in reporter cells and in a bronchial epithelial cell line derived from a cystic fibrosis patient. These data suggest that the structure of PTC124 may be further optimized to mediate more efficient suppression at some PTCs [86]. In addition, a novel PTC124 analog, PTC-414, was recently described to be able to induce as well as PTC124 functional rescue of REP1 in human choroideremia fibroblasts and the nonsense mediated zebrafish model [87]. In a recent study, the nucleoside analog clitocine was identified as a potent readthrough agent [88]. Incorporation of clitocine into RNA at the third position of a premature termination codon results in the suppression of nonsense mutations in cells harboring p53 nonsense alleles. In these cells, full length p53 was restored and was functional in the transcriptional activation of p53 regulated genes. This novel mechanism represents an alternate therapeutic modality for treatment of cancers and genetic diseases caused by nonsense mutations [88]. Overall, these results indicate that a wide chemicals repertoire, involving different action mechanisms, may be available for treatments of cancers and genetic diseases caused by nonsense mutations.

## 4. Negative Modulation of Nonsense Mediated mRNA Decay

Introduction of a PTC within an open reading frame can generate the synthesis of a truncated protein that could be un-functional, partially functional, or aberrant for a gained negative-dominant function. In the latter case, NMD has a surveillance function preventing the synthesis of harmful protein by accelerating the turnover of the mRNA harboring the PTC. In doing this, even when the truncated protein still retains partial function, NMD precludes its expression, worsening the disease phenotype. In all cases, NMD decreases the expression of mRNA containing PTCs, limiting the potential of the nonsense suppression therapy [10,22]. The functional consequences of PTC on gene expression are schematically exemplified in Figure 1. In principle, an efficient NMD would alleviate disorders potentially caused by anomalous truncated protein, whereas inhibition of NMD would increase the pool of mRNA that could be translated in diseases caused by a lack of a specific function (see the scheme in Figure 1B). The efficiency of NMD is individually variable within the same inherited disease and can have critical consequences on the severity of the disease [89,90,91,92,93]. A clear example of a personal level of NMD is represented by CF patients carrying the nonsense mutation W1282X in the CFTR gene, whose derived nasal epithelial cells present different levels of mRNA encoding that mutation [92]. In these cells, the readthrough response to gentamicin is correlated with the abundance of the CFTR W1282X harboring the transcript with no response in cells with the lowest CFTR transcript. Consistently, negative modulation of NMD in cells from patients unresponsive to gentamicin alone rescued the nonsense suppression. More recently a similar approach was used in vivo with a mouse model of the lysosomal storage disease mucopolysaccharidosis I-Hurler (MPS I-H) [58]. Keeling and co-workers showed that aminoglycoside and the NMD inhibitor NMDI-1, when concomitantly administered to mice carrying a PTC in the *Idua* locus, resulted in increased enzyme function compared to aminoglycoside used as a single readthrough agent. In addition to NMDI-1, other NMD inhibitors, have been described as effective in genetic diseases and cancer caused by nonsense mutations [94]. NMD was found negatively modulated by 5-azacytidine, an analogue of the naturally occurring pyrimidine nucleoside cytidine, a drug that was already approved for the treatment of myelodysplastic syndrome and myeloid leukemia [95]. Among the repurposed drugs, amlexanox is unique in that combines both readthrough and NMD attenuation properties and was identified in a dedicated screening of compounds already used in the clinic for other purposes [85]. This approach, i.e., searching among already known compounds those with readthrough and possible NMD modulating properties, is of advantage in that it avoids the long optimization and development process required for access to the clinic. This would, hopefully, facilitate the use of drugs like amlexanox in nonsense suppression therapy. Finally, NMD was found to be modulated by rapamycin, a naturally occurring macrolide known to be an inhibitor of cap dependent mRNA translation [96]. The emerging picture of this study is that mammalian cells can regulate NMD efficiency of mRNA loaded with polyribosomes as a function of cellular need.

## 5. β-Thalassemia

In the last few years, nonsense suppression therapy has been approached for a variety of genetic diseases based on nonsense mutations, including lysosomal storage diseases (LSDs) and genetic eye disorders. The first two diseases have been recently reviewed respectively [22,23]. Here, we focus on β-thalassemia, one of the most widespread autosomal recessive disorders worldwide [97] caused by the reduced (β^−^) or absent (β^0^) synthesis of the β-globin chains of adult hemoglobin (HbA).

The β-globin gene is located in the distal portion of the short arm of chromosome 11, within the β-globin gene cluster. It extends for 1.6 Kb and contains three exons, two introns, and two sequences in the flanking 5′ and 3′ defined as untranslated regions (UTRs) [98]. Its expression is regulated by an upstream promoter, containing TATA (TATA box) and CAAT (CAAT box) sequences and duplicated CACCC sequences (CACC distal and proximal boxes) [97]. A major regulatory region, containing also a strong enhancer, maps 50 Kb from the β-globin gene. This region, called the locus control region (LCR), contains five (HS-1 to HS-5) erythroid specific DNAse hypersensitive sites (HSs), which are the hallmark of DNA–protein interaction. Each HS site is constituted of a combination of several DNA motifs interacting with transcription factors, among which the most important are GATA-1, nuclear factor erythroid 2, and erythroid Krüppel-like factor [97].

### 5.1. Nonsense Mutations and Nonsense Mediated mRNA Decay in β-Thalassemia

Nonsense mutations are among the most important alterations of the β-globin gene in β-thalassemia patients and are based on a change of an amino acid codon to a premature termination codon (PTC). For instance, in β^0^39-thalassemia, the CAG (glutamine) codon of the β-globin mRNA is mutated to the UAG stop codon [98,99], leading to premature translation termination and to mRNA destabilization through the NMD mechanism [100,101,102,103]. The activation or not of the NMD mechanism impacts the β-thalassemia phenotype. As summarized by Peixeiro et al. [104], β-globin nonsense mutations located in the 3′ region of exon 1 (for example, at codon 26) and within the 5′ region of exon 2 (at codons 36, 60/61, 75, and 82) promote NMD. In contrast, mRNAs bearing PTCs towards the 3′ end of exon 2 (at codons 88, 91, 95, 98, 101, and 103) and those with PTCs in exon 3 (at codons 106, 107, 114, 121, and 141) are all NMD resistant [102,103,104,105,106]. The NMD mechanism usually requires a minimum distance between the nonsense mutation and the final exon-exon junction, but on β-globin transcripts, the required maximum distance appears to be considerably less important. Indeed, a β-globin mRNA with a nonsense mutation at codon 39 [102,107] elicits NMD machinery, even when the distance between the PTC and the 3′ exon-exon junction is increased from the normal 180 nt up to 654 nt [104,108].

The disease is inherited in a recessive mode, and heterozygote subjects are asymptomatic, when the PTC is located at a position activating NMD. In this case, the NMD degradation of the defective β-globin transcript produces a reduced synthesis of the truncated protein [104]. If the PTC is located at a position that does not induce NMD (at less than 55 nt upstream of the last exon-exon junction or at the 5′-part of exon 3), the small quantity of synthesized truncated product could be efficiently degraded by the proteolytic system of the red blood cell, and the relative heterozygotes will be asymptomatic [106]. If the PTC is located further downstream at exon 3 of the β-globin mRNA, NMD is also not activated. In this condition, the mutant β-globin mRNAs, encoding for the truncated protein, are long enough to overload the cellular proteolytic system [104]. The excessive accumulation of the truncated products, as well as, free α-globin chains causes the precipitation of insoluble globins, leading to cellular toxic effects. This condition, called thalassemia intermedia, is correlated with a symptomatic form in heterozygotes and a dominant modality of inheritance [104,109]. In Table 1 is reported a list of PTCs on β-globin eliciting or not eliciting NMD.

### 5.2. Readthrough Approach for β-Thalassemia

In β-thalassemia, the absence or reduced synthesis of β-globin chains causes severe chronic anemia and treatment related complications. Patients are usually treated with chronic blood transfusions and iron chelation therapy. Alternatively, bone marrow transplantation has been proposed. Gene therapy is still not currently available. In addition, novel strategies such as the ribosomal readthrough could introduce new hopes for the development of a pharmacologic approach to cure β-thalassemia caused by PTC, such as β^0^-39-thalassemia.

Recently, Salvatori et al. [110] reported the development of a novel experimental system suitable to screen potential modifiers of the biological consequences of stop mutations. This system was generated using two lentiviral constructs, one containing the human normal β-globin gene and the other containing the β°39-thalassemia globin gene, both under the control of the β-globin promoter and an LCR cassette. These vectors were transfected to K562 cells and several isolated K562 cell clones, expressing either the normal β-globin or the β°39-thalassemia globin genes at different levels. Then, the system was evaluated to detect the readthrough molecules such as aminoglycosides [110].

In another article, Salvatori et al. [111] demonstrated that aminoglycoside geneticin (G418) was able to induce production of β-globin in cells carrying β-globin genes with the β°39-thalassemia mutation, by the readthrough mechanism, leading to translation of β°39-globin mRNA and ultimate production of HbA [112]. This was reproducibly obtained using K562 cell clones carrying β°39-globin genes previously described [110].

Moreover, an efficient production of β-globin by β°39-globin mRNA was reported in erythroid precursor cells isolated from six homozygous β°39-thalassemia patients and treated with erythropoietin (EPO) with or without G418. Using G418, the conversion of a high proportion of these cells from being negative for β-globin chain synthesis to β-globin producing cells was observed by both FACS and HPLC analyses [111]. Obviously, this finding is far from obtaining a full restoration of HbA content in homozygous β°39-thalassemic cells, due to the fact that the β°39-globin mRNA is present in very low amounts, even if a partial increase of HbA might be beneficial in patients carrying selected genotypes (for instance β°/β^+^) or when this approach is carried out in combination with other treatments, such as hydroxyurea, as inducers of HbF production [113] or NMD inhibitors [112,114,115]. For instance, silencing RNAs against SMG-1 and Upf1 strongly inhibits NMD with a mechanism of action clearly different from G418 mediated effects [114,115,116]. In addition, inhibition of NMD can be reached under hypoxic conditions, as suggested by Gardner [112].

Recently, Kar et al. [117] investigated oligonucleotides targeting HBB mRNA downstream of the premature stop codon as novel and potential readthrough molecules for the treatment of β-thalassemia, producing a full length β-globin protein and opening a novel opportunity in the field of readthrough strategies of PTCs.

Finally, the current clinical trials under investigation for β-thalassemia are relatives of gene therapies, HbF inducers, iron chelators, but not for readthrough molecules, which should be considered as potential and alternative approaches to be deeply investigated for this hemoglobinopathy. However, the possible application of readthrough molecules to β-thalassemia is clearly mentioned in some granted patents (such as USP-9255088, USP-9358246, and USP-10398718) in which the possible application of the invented compounds for personalized therapy of β-thalassemia is clearly proposed [118,119,120].

Moreover, β-thalassemia is characterized by ineffective erythropoiesis (IE) and anemia, so novel approaches should restore the tissue oxygenation producing adequate and functional red blood cells. From this point of view, the iron reduction can decrease erythroid cells’ production with abnormal Hb and improve erythropoiesis. In particular, novel molecules targeting iron (e.g., mini-hepcidin agonists, serine protease in combination with iron chelators, apotransferrin) can be useful to improve IE [118,119,120,121] and should be investigated in combination with readthrough inducers.

## 6. Conclusions and Perspectives

Since the original evidence that nonsense suppression approach could provide a means to restore the synthesis and the function of CFTR, inactivated by a nonsense mutation, many studies have explored the feasibility of the nonsense suppression approach through numerous genetic diseases caused by PTC [10,24,47]. The possibility to extend the therapeutic principle to most if not all genetic diseases associated with nonsense mutations, including several forms of cancer, has presented new expectations to cure different diseases by using the same therapeutic based on the stop codon readthrough mediated by small compounds. Several variables are likely to play a role in the success of the nonsense suppression therapy. First is the availability of novel safe compounds effective at inducing readthrough with no side effects. In this context, the possibility to re-design the aminoglycosides, including the new entry gentamicin B1, so as to minimize the toxic moieties while saving the readthrough properties, may soon provide the drug for the long term treatments required for the cure of these severe diseases. A great advantage would come by using a much lower concentration of aminoglycosides when co-administered with enhancer molecules, of the type of CDX compounds, once proven to be safe. Second, assessment of the therapeutic threshold for each disease, in terms of the minimal product production needed to alleviate the relevant phenotype, would help in designing a proper treatment strategy. Third, a combined therapy should be evaluated when mRNA is particularly destabilized by NMD. The discovery of amlexanox, combining readthrough and NMD inhibition properties, suggests that future screenings could be addressed to this category of compounds. Inhibition of NMD as a therapeutic approach may result in various effects and is achieved by using drugs [94]. In this context, strategic targeting of genes encoding components of the complex NMD machinery by clustered regularly interspaced short palindromic repeats (CRISPR/Cas9) technologies has been evaluated [119,120,121,122]. However, recent studies provided evidence that NMD is not only involved in the mRNA surveillance pathway, but constitutes a post-transcriptional control of gene expression and is at the crossroads of many cellular functions [13,37,120,121,122,123,124,125,126]. Thus, the catalogue of the mRNA target of NMD has been extended to hundreds of physiological transcripts encoding no PTC [38,39]. In this respect, although being promising for the treatment of many nonsense mutation related diseases, modulation of NMD is an approach that must be taken with caution. In addition, NMD efficacy is variable and has an impact on the clinical outcome of PTC. Interestingly enough however, a powerful method to study NMD variability by a single molecule is now available and is revealing rules governing the fate of mRNA harboring PTCs [124,125,126,127]. Moreover, the impact of introducing PTCs at the human genome level has been, very recently, approached by developing a resource, NMDetective, for genome wide prediction of NMD efficacy in human and mouse models [125,126,127,128]. Results have shown that (a) NMD often exacerbated the disease, (b) failure to trigger NMD can be a cause of ineffective gene inactivation by CRISPR/Cas9 gene editing, and (c) inhibiting NMD may be effective at enhancing cancer immunotherapy. These important achievements on key mechanisms influencing NMD suggest that identifying the PTC for each patient is crucial in the decision about the most appropriate therapeutic approach. In particular, when NMD leads to a worsened phenotype, then the well tolerated NMD inhibitors could be useful for a broad range of genetic diseases.

Thalassemia is one the most important health problems in developing countries mainly because of the absence of genetic counseling and prenatal diagnosis [126,127,128,129]. The pharmacological therapy of β-thalassemia is expected to be crucial for several developing countries, unable to sustain efficiently the high cost clinical management of β-thalassemia patients requiring a regular transfusion regimen, chelation therapy, and advanced hospital facilities. Alternative and promising therapeutic approaches such as gene therapy and bone marrow transplantation are expected to be useful only for a minority of patients, based on specific biological/genetic parameters and with the economic possibility to afford these therapies [118,127,128,129,130]. Therefore, the discovery of molecules exhibiting the property of inducing β-globin, such as readthrough compounds, is of great interest and represents a hope for several patients, whose survival will depend on the possible use of drugs rendering blood transfusion and chelation therapy unnecessary. In this respect, the rapid and inexpensive dual fluorescence screening system based on the yeast *S. cerevisiae*, recently developed, could be useful for the identification of novel small readthrough compounds and/or NMD modulators [128,129,130,131].

In conclusion, while significant progress has been made in the development of a therapeutic strategy for the treatment of disparate genetic diseases and various forms of cancer, combining nonsense mutations, clinical issues still remain below expectations. In this context, we are aware that the use of readthrough mediating compounds is often associated with many risks and difficulties related to not only drug toxicity to the cell and to the organism, but also to the lack of certainty about the identity of amino acids decoded under the readthrough treatment. Nevertheless, considering the extreme severity of many PTC caused genetic diseases and considering that even a small response to the therapy could make the difference, any effort to improve the readthrough therapeutic approach should be produced. As an example, the small and safe molecule ataluren has been recently used in CF cells in combination with caffeine, an attenuator of NMD, and found to increase the recovery of a functional full-length CFTR protein [129,130,131,132]. Hopefully, such results might be reproduced in the clinic.

A recent study, combining genomic, proteomic, and biochemical data, demonstrated that many common nonsense variants do not necessarily result in loss of protein production and provided experimental evidence for the existence of gene rescue mechanisms [130,131,132,133]. In this respect, it is likely that in the near future, increased knowledge about the human genome’s plasticity will play a key role in understanding how the general genetic background establishes the individual threshold in response to the nonsense suppression therapy of a given genetic disease.

## Figures and Tables

**Table 1:** β-thalassemia nonsense mutations. For each mutation, sense codon, stop codon, nucleotide substitution, relative frequency (indicating the mutation occurrence in a specific country or geographic area based on all the β-thalassemia patients and obtained from the HbVar database and NMD activation are reported.

Nonsense Mutation	Sense/Nonsense Codon	Nucleotide Substitution	Frequency	NMD Activation	Reference
β°15	TGG/TAG	G→A	Bangladesh 10%	NO	97
β°15	TGG/TGA	G→A	Portugal 11.79% Russia 6.45%	NO	98
β°17	AAG/TAG	A→T	Thailand 18.56% China 14.1%	NO	98
β°22	GAA/TAA	G→T	La Réunion (one case)	NO	98
β°26	GAG/TAG	G→T	Thailand 0.12%	YES	99
β°35	TAC/TAA	C→A	Thailand 1.22%	YES	99
β°37	TGG/TAG	G→A	Afghanistan	YES	100
β°37	TGG/TGA	G→A	Arab countries 18.6%	YES	101
β°39	CAG/TAG	C→T	Italy 66.84%; Argentina 47.06%; Portugal 34.9%; England 34.78%	YES	102
β°43	GAG/TAG	G→T	Singapore 0.75%; Thailand 0.37%	YES	103
β°59	AAG/TAG	A→T	Italian-American Family	YES	104
β°61	AAG/TAG	A→T	USA (one case)	YES	105
β°90	GAG/TAG	G→T	Japan 13.8%	YES	99
β°112	TGT/TGA	T→A	Slovakia (one case)	YES	96
β°121	GAA/TAA	G→T	Czechoslovakia 11.83% England 13.04%	YES	106
β°127	CAG/TAG	C→T	England (one case)	NO	107

## Abbreviations

APC	adenomatous polyposis coli
CF	Cystic Fibrosis
CFTR	cystic fibrosis trans-membrane conductance regulator
DMD	Duchenne muscular dystrophy
HbA	Adult Hemoglobin (HbA)
HBE	human bronchial epithelial
HSs	hypersensitive sites
LCR	locus control region
LSD	lysosomal storage diseases
MPS I-H	mucopolysaccharidosis I-Hurler
NMD	Nonsense mRNA-mediated decay
PTCs	premature termination codons
UTRs	untranslated regions
